# Molecular development of chondrichthyan claspers and the evolution of copulatory organs

**DOI:** 10.1038/ncomms7698

**Published:** 2015-04-14

**Authors:** Katherine L. O'Shaughnessy, Randall D. Dahn, Martin J. Cohn

**Affiliations:** 1 Department of Molecular Genetics and Microbiology, UF Genetics Institute, University of Florida, PO Box 103610, Gainesville, Florida 32610, USA; 2 7322 Countrywood Lane, Madison, Wisconsin 53719, USA; 3 Howard Hughes Medical Institute and Department of Biology, University of Florida, PO Box 103610, Gainesville, Florida 32610, USA

## Abstract

The earliest known vertebrate copulatory organs are claspers, paired penis-like structures that are associated with evolution of internal fertilization and viviparity in Devonian placoderms. Today, only male chondrichthyans possess claspers, which extend from posterior pelvic fins and function as intromittent organs. Here we report that clasper development from pelvic fins of male skates is controlled by hormonal regulation of the *Sonic hedgehog* (*Shh*) pathway. We show that Shh signalling is necessary for male clasper development and is sufficient to induce clasper cartilages in females. Androgen receptor (AR) controls the male-specific pattern of *Shh* in pelvic fins by regulation of *Hand2*. We identify an androgen response element (ARE) in the *Hand2* locus and present biochemical evidence that AR can directly bind the *Hand2* ARE. Together, our results suggest that the genetic circuit for appendage development evolved an androgen regulatory input, which prolonged signalling activity and drove clasper skeletogenesis in male fins.

## Abstract (web summary)

Claspers are copulatory organs found in male cartilaginous fishes. Here, the authors show that androgen receptor signalling maintains the Shh pathway to promote clasper development in male skates and suggest the importance of hormonal regulation in the evolution of male copulatory organs.

## Graphical abstract

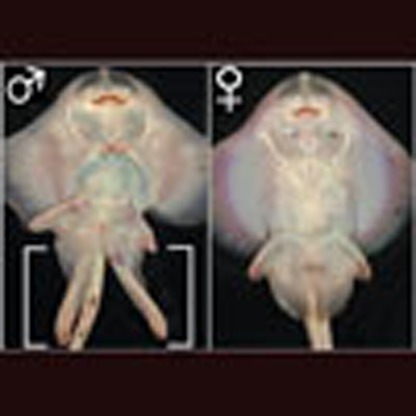

The origin of vertebrate copulation, internal fertilization and viviparity can be traced back ∼380 million years to the most basal gnathostome group, the placoderms^1^,^2^,^3^. Fossils of these armored fishes show evidence of embryos internally, and adults have intromittent organs in the form of paired claspers that resemble those found in male chondrichthyans (sharks, skates, rays and chimaeras) but articulate at more posterior positions^1^,^2^,^3^. Claspers in chondrichthyans are specialized elongations on the posterior side of male pelvic fins that are used for sperm transfer during copulation. Little is known about the mechanisms of clasper evolution, or why only males undergo this fin modification. Here we investigate the molecular basis of clasper development in the little skate, *Leucoraja erinacea*, and show that AR directly regulates *Hand2* to maintain *Shh* signalling in pelvic fins of male embryos. This prolonged phase of *Shh* expression at the posterior margin of male pelvic fins sustains the appendage development circuit and promotes localized outgrowth of claspers.

## Results

### Claspers form during a late phase of pelvic fin development in males

**Figure 1:**
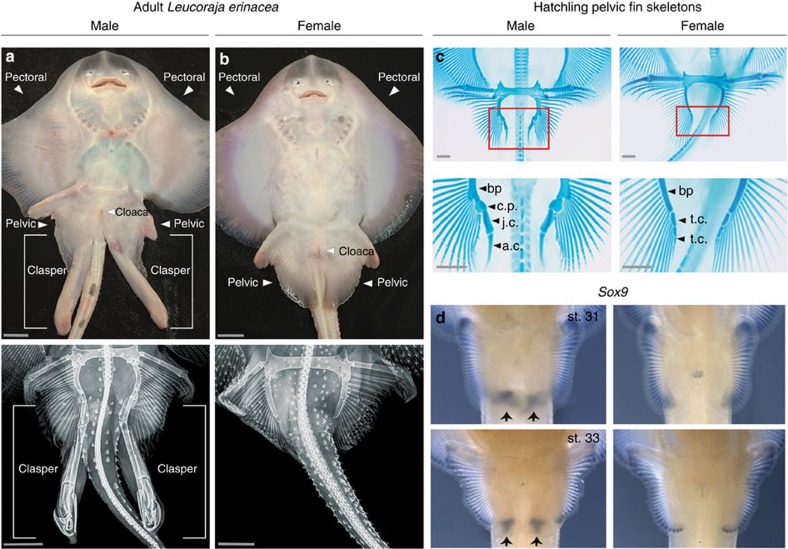
Morphology and development of *L. erinacea* claspers. (**a**,**b**) Ventral view of sexually mature male (**a**) and female (**b**) skates. In these panels the scale bar, 2.5 cm. Lower panels show corresponding X-rays of the male and female pelvic fins with scale bars, 4 cm. (**c**) Alcian blue staining of hatchling pelvic fins. The posterior skeletal elements of males and females are labelled as follows: bp, basipterygium; t.c., terminal cartilages; c.p., covering plate; j.c., junctional cartilages; a.c., axial cartilage. Scale bars, 1 mm. (**d**) *Sox9* expression in developing male (left) and female (right) pelvic fins at stages 31 and 33.

The pelvic fin skeletons of male and female skates, similar to other chondrichthyans, consist of a propterygium, basipterygium and radials; however, only males develop an elongated clasper, or myxopterygium^4^ (Fig. 1a,c and Supplementary Figs 1 and 2b). In females, the only skeletal structures posterior to the basipterygium are small nodular elements known as terminal cartilages (Fig. 1b,c; Supplementary Figs 1 and 2b). To investigate how these sex differences in pelvic fin morphology arise during embryogenesis, we compared pelvic fin development in males and females of the little skate. Fin buds of males and females are indistinguishable at early stages of development (through stage 29; Supplementary Fig. 1b and Supplementary Table 1). The earliest evidence of clasper development appears at stage 30, when males undergo expansion of the posterior part of the pelvic fin bud (Supplementary Fig. 1b,c). The chondrogenic transcription factor *Sox9*, which is expressed in distal radials in both sexes at stage 30, is then activated in the posterior pelvic fin bud mesenchyme only in males (Fig. 1d and Supplementary Fig. 2a). By stage 31, a discrete clasper bud has emerged posteriorly on male pelvic fins (Supplementary Fig. 1b,c), and alcian blue staining reveals the formation of an elongated cartilage condensation posterior to the basipterygium (Supplementary Fig. 2b). *Sox9* expression persists in the clasper bud mesenchyme, and clasper cartilages continue to condense and differentiate posteriorly for the duration of development (Fig. 1d and Supplementary Fig. 2). By hatchling stage, the major components of the clasper skeleton, including the short junctional cartilages, the covering plate and the long axial and terminal cartilages, have differentiated (Fig. 1c). Female pelvic fin buds do not undergo posterior expansion (Supplementary Fig. 1b); instead, only small chondrogenic condensations form at the tip of the basipterygium, where they differentiate into the terminal cartilages (Fig. 1c and Supplementary Fig. 2b).

### The appendage outgrowth circuit is maintained in male clasper buds

**Figure 2:**
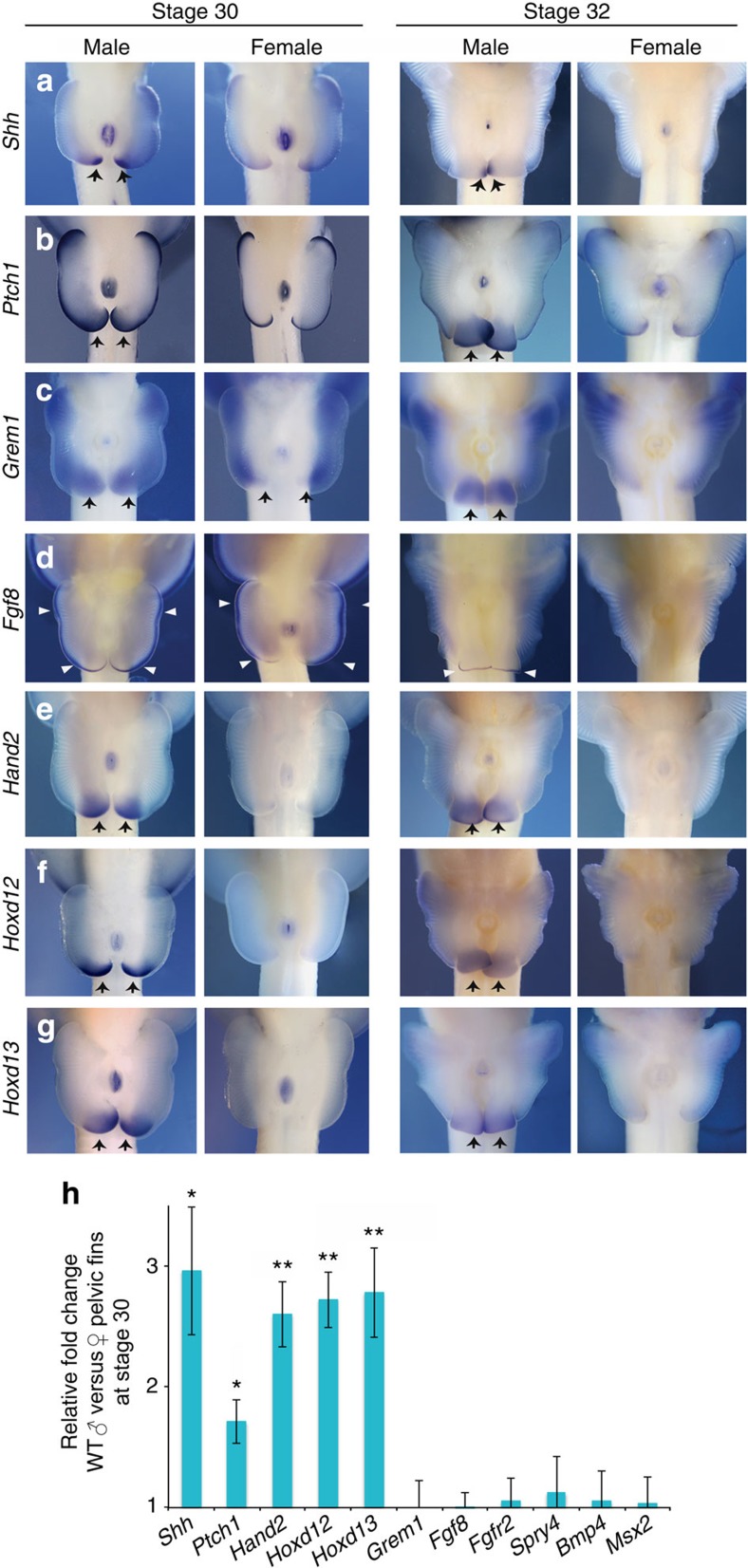
Sexually dimorphic gene expression during male clasper development. (**a**–**g**) *In situ* hybridization of embryonic pelvic fins during clasper initiation (stage 30) and advanced clasper morphogenesis (stage 32). Black arrows mark mesenchymal expression and white arrows mark epithelial expression. (**h**) Relative gene expression in male versus female pelvic fins at clasper initiation. Fold changes were determined by the ΔΔ*C*_t_ method and error bars represent±s.e.m. Asterisks denote significant differences, where one is *P*<0.05 and two is *P*<0.01. *N*=4 biological replicates and 3 technical replicates for both male and female embryos.

Sustained posterior outgrowth and skeletogenesis in male pelvic fins suggested that the genetic circuit that regulates fin development could remain active for an extended period in male skates. Two signalling regions in fin (and limb) buds establish this circuit; the mesenchymal zone of polarizing activity (ZPA) controls anteroposterior patterning, and the epithelial apical ectodermal ridge (AER) controls proximodistal outgrowth. A positive feedback loop between Shh and Gremlin1 (Grem1) in the posterior mesenchyme and Fgfs in the AER coordinates limb patterning with outgrowth^5^. To determine whether signalling is sustained in male pelvic fins beyond the stage when the circuit is inactivated in females, we compared spatial, temporal and quantitative expression patterns of the constituent genes between sexes. Whole-mount *in situ* hybridization showed that *Shh* is expressed posteriorly in male and female pelvic fins at stage 29; however, only male fins continue to express *Shh* at stage 30 (Fig. 2a and Supplementary Fig. 3a)^6^. *Shh* expression persisted in the posterior region of male pelvic fin buds for ∼4 weeks (through stage 32; Supplementary Table 1) after expression had terminated in females (Fig. 2a). Similarly, the Shh target gene *Ptch1*, a readout of Shh signalling^7^, continued to be expressed in the posterior pelvic fin mesenchyme of males but not females, indicating that Shh signal transduction remained active for approximately a month longer in males (Fig. 2b and Supplementary Fig. 3b).

The finding that Shh signalling persists in male clasper buds raised the possibility that other signalling regions also may be maintained in male pelvic fins. To determine whether outgrowth of the clasper bud is associated with sexually dimorphic maintenance of AER factors, we examined expression of *Grem1*, the mesenchymal factor that mediates Shh signalling to the AER, and *Fgf8*, which is expressed throughout the AER of fins and limbs and signals back to the mesenchyme. Males and females show similar levels and patterns of *Grem1* and *Fgf8* expression at stages 29 and 30 (Fig. 2h); however, over the next two stages, *Grem1* becomes enriched in the clasper-forming region of male fin bud mesenchyme (Fig. 2c and Supplementary Fig. 3c). In addition, *Fgf8* expression regressed from anterior to posterior, eventually disappearing in females at stage 32, while males maintained a small domain of *Fgf8* expression in the clasper bud, in an AER-like pattern complementary to the *Shh* domain (Fig. 2d and Supplementary Figs 3d and 4a). Analysis of *Sprouty4* (*Spry4*), a sensor of Fgf signalling^8^, revealed similar distal patterns of expression in male and female pelvic fins through stage 30 (Fig. 2h); however, posterior expression is maintained only in males, consistent with prolonged Fgf signalling (Supplementary Fig. 4d). Because Gremlin sustains Fgf expression in tetrapod limbs by antagonizing the activity of Bmp4 posteriorly^5^,^9^, we tested whether this component of the circuit operates during clasper formation. *Bmp4* and its downstream effector, *Msx2*, are expressed similarly in early fins of males and females (through stage 30); however, a sexually dimorphic pattern appears from stage 31. Male fins show enriched expression posteriorly, and by stage 32, expression is found only in the clasper-forming region (Supplementary Fig. 4b,c). Together, these results show that the gene regulatory network that drives appendage development remains active in the clasper-forming region of male fins. The finding that male-specific maintenance of *Shh* precedes the dimorphic patterns of *Grem1* in the mesenchyme and *Fgf8* in the distal ectoderm suggests that sustained activity of the *Shh* is an early step in clasper bud development.

Posterior expression of *Shh* in mouse limb buds has been shown to be controlled by transcription factors encoded by *Hand2* and 5′ *HoxD* genes^10^,^11^,^12^,^13^. Hand2 and Hoxd proteins can directly activate an appendage-specific enhancer of *Shh* known as the ZRS (ZPA regulatory sequence)^14^, which is conserved across gnathostomes, including skates^6^. Therefore, we investigated whether prolonged expression of *Shh* in the clasper-forming region of male fins is associated with temporal changes in expression of *Hand2*, *Hoxd12* and *Hoxd13*. Transcripts of all three genes were detected in the posterior mesenchyme of male but not female pelvic fins at stage 30 (Fig. 2e–g). Quantitative comparison of mRNA levels by quantitative reverse transcriptase–PCR (qRT–PCR) in male and female pelvic fins at the onset of clasper development (st. 30) confirmed sexually dimorphic expression of *Hand2*, *Hoxd12* and *Hoxd13*, as well as *Shh* and *Ptch1* in male fins (Fig. 2h). By contrast, *Grem1, Fgf8, Fgfr2, Spry4, Bmp4* and *Msx2* were not yet different between sexes at stage 30 (Fig. 2h). Expression of *Hand2*, *Hoxd12* and *Hoxd13* persisted in the clasper buds of male fins through stage 32 but was undetectable in female pelvic fins (Fig. 2 and Supplementary Fig. 3), indicating that the prolonged period of *Shh* expression in the posterior region of male fins is associated with sustained activity of transcription factors known to directly regulate its expression in the ZPA^14^.

### SHH is necessary and sufficient for clasper development

**Figure 3:**
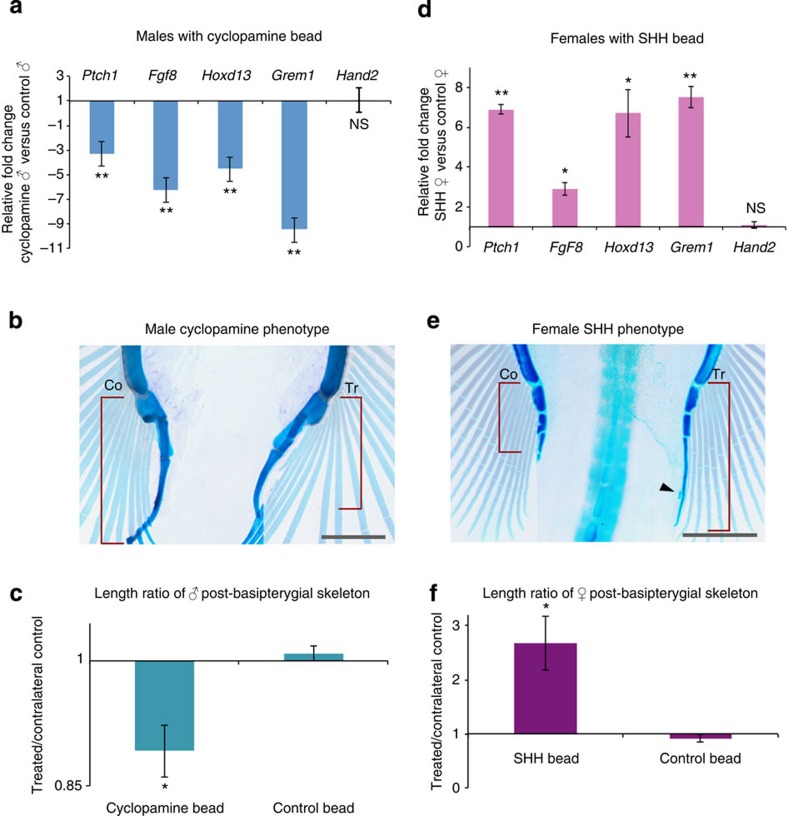
Shh is necessary and sufficient for clasper formation. In all statistical analyses, error bars represent±s.e.m., and asterisks denote significant differences, where one indicates *P*<0.05 and two indicate *P*<0.01. (**a**) Relative gene expression values in male fin buds implanted with a cyclopamine bead versus those implanted with control bead. *N*=4 cyclopamine males and *N*=4 control males (three technical replicates of each) used in qRT–PCR expression analysis. (**b**) Clasper skeletons of hatchling male after implantation of a cyclopamine bead into right fin at stage 30. Note that a contrasting mask is used to highlight the ectopic structures without removing the rest of the fin radials. Right fin (Tr, treated) shows truncated clasper skeleton induced by cyclopamine, whereas the left side shows the untreated fin (Co, contralateral). Scale bar, 2 mm. (**c**) Quantification of clasper length in cyclopamine-treated animals relative to controls. Asterisk indicates a significant difference (*P*<0.05). *N*=8 cyclopamine-treated males and *N*=4 control males. (**d**) Relative gene expression values in female fins receiving a SHH bead (*N*=4) or a control bead (*N*=5). (**e**) Posterior fin skeleton of hatchling female after implantation of SHH bead into right fin. Note the formation of elongated clasper-like cartilage (red bracket) and accessory cartilage (black arrow) on the treated (right) side. Scale bar, 2 mm. (**f**) Quantification of post-basipterygial cartilage length in SHH-treated and control female fins. *N*=6 SHH-treated females and *N*=5 control females. Asterisk indicates significant difference (*P*<0.05).

To determine whether the extended phase of *Shh* activity is required for clasper development, we implanted carrier beads loaded with cyclopamine, an inhibitor of hedgehog signal transduction^15^, into the posterior region of male skate pelvic fins at stage 30. Twenty-four hours after bead implantation, *Ptch1*, *Fgf8* and *Hoxd13* were significantly downregulated in cyclopamine-treated fin buds (Fig. 3a and Supplementary Fig. 5). Although *Hoxd13* responded to cyclopamine, *Hand2* was unchanged, suggesting that the structure of the circuit is conserved between skate claspers and tetrapod limbs, in which *Hoxd13* is both upstream and downstream of *Shh*, but *Hand2* acts only upstream of *Shh*^12^,^13^. We then examined the effects of cyclopamine on development of the clasper skeleton. Quantification of the length of post-basipterygial skeletal elements in hatchlings revealed that antagonism of SHH signalling by cyclopamine resulted in significant reduction in the clasper skeleton (*P*=0.017; Fig. 3b,c). By contrast, control beads had no effect on clasper formation.

Having shown that SHH is required for normal clasper development in males, we then asked whether SHH alone is sufficient to maintain this genetic circuit and induce clasper development in females. Implantation of SHH-loaded beads into the posterior region of female fins at stage 30 resulted in significant upregulation of *Ptch1*, *Fgf8*, *Hoxd13* and *Grem1* (Fig. 3d and Supplementary Fig. 5). As with the cyclopamine experiments described above, *Hand2* did not respond to SHH (Fig. 3d). Analysis of female fin skeletal patterns after ∼10 weeks of development revealed the presence of clasper cartilages posterior to the basipterygium of SHH-treated fins (Fig. 3e). Measurements of the cartilages that developed posterior to the basipterygium revealed that SHH increased their length by 268% relative to the contralateral fin (*P*=0.024; Fig. 3f). By contrast, fins receiving control beads were not significantly different from contralateral fins (Fig. 3f). Thus, prolonged SHH activity is sufficient to sustain the feedback loop in the posterior region of the pelvic fin bud and to induce formation of clasper skeletal elements in female skates.

### Androgen receptor (AR) regulates *Shh* expression in clasper buds

**Figure 4:**
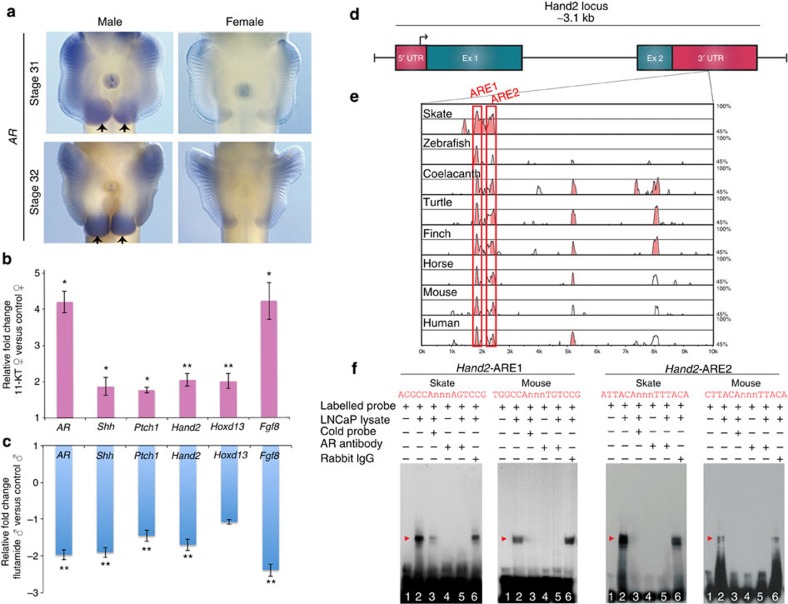
Androgen receptor regulation of the fin development circuit in skate pelvic fins. (**a**) *In situ* hybridization of *AR* in male and female pelvic fins. Note the strong expression in the developing claspers (arrows). (**b**) Relative levels of gene expression in 11-KT-treated female fins (*N*=5) versus controls (*N*=4). (**c**) Relative gene expression in male fins after functional inactivation of AR by flutamide. Fold changes were determined by comparison of flutamide-treated (*N*=7) versus control male (*N*=5) pelvic fins. Error bars in **b**,**c** represent±s.e.m.; asterisks denote significant differences where one indicates *P*<0.05 and two indicate *P*<0.01. (**d**) Schematic diagram of the *Hand2* locus in *L. erinacea*. (**e**) VISTA plot of a portion of the 3′ UTR of *Hand2* in eight vertebrates, with elephant shark as the reference sequence. The first two peaks (boxed) contain conserved AREs (labelled ARE1 and ARE2). (**f**) Gel shift assay demonstrates binding of *Hand2*-ARE1 and ARE2 by androgen receptor protein. Red arrows denote shifts indicative of protein:DNA complexes. In all EMSA blots, Lane 1 is free probe only, Lane 2 is labelled probe and LNCaP lysate, and Lane 3 is cold competition assay (observed shift is outcompeted with excess unlabelled probe). Lane 4 is labelled probe and antibody only (no interaction). Lane 5 is labelled probe, LNCaP lysate and antibody; the specific block shift in Lane 5 confirms that the shift in Lane 2 was specific to formation of an AR:DNA complex. Lane 6 is an additional control using Rabbit IgG in place of the AR antibody; the persistence of the shift provides further support that the the block shift in Lane 5 is specific to AR.

A number of the genes expressed in skate clasper buds also have roles in mammalian genitourinary development, where they are regulated by androgens^16^,^17^,^18^. Moreover, our previous studies showed that the ratio of androgen to oestrogen signalling underlies differential expression of genes involved in sexually dimorphic digit growth in mice^19^. To determine whether androgen signalling plays a role in male chondrichthyan clasper development, we cloned skate AR and investigated its expression in male and female pelvic fins. *AR* expression was detected in the pelvic fin buds of both sexes, but expression was enriched in the posterior region of the male fin bud mesenchyme (Fig. 4a). *AR* expression persisted in the clasper bud through stage 32, with the strongest signal observed in the clasper-forming region (Fig. 4a). To test directly whether differential androgen activity in males and females could underlie sexually dimorphic gene expression in the posterior region of pelvic fins, we performed functional manipulations of androgen signalling in skate embryos at stage 30, when male clasper development is initiated. AR activity was antagonized in male embryos by treatment with flutamide, which directly binds AR and inhibits signalling^20^, and gene expression was examined after 96 h (see Methods). Inhibition of AR function in male embryos resulted in significant downregulation of *Hand2, Shh, Ptch1*, *Fgf8* and *AR* itself in male pelvic fins (Fig. 4c).

In a reciprocal experiment, we investigated whether activation of AR signalling in female fins exposed to androgen could be sufficient to induce a clasper-like pattern of gene expression. Female skate embryos treated with 11-KT, the major androgen in fishes^21^, led to a more than fourfold increase in *AR* transcription and induction of male patterns of gene expression in the pelvic fins (Fig. 4b). Specifically, *Hand2, Hoxd13, Shh, Ptch1* and *Fgf8* were significantly upregulated in pelvic fins of 11-KT-treated females relative to controls (Fig. 4b). It is noteworthy that androgen resulted in sustained expression of these genes after transcription would normally be terminated in the posterior region of female pelvic fins, but did not lead to ectopic activation elsewhere in fins. This suggests that during normal development of male pelvic fins, AR functions to maintain gene expression domains that were initiated during early stages of fin development, and this sustains outgrowth of the clasper-forming region of the pelvic fins.

### AR binds androgen response elements in Hand2, a direct regulator of *Shh*

The results of AR modulation led us to investigate whether *Hand2* could be a direct target of AR and, therefore, mediate AR induction of *Shh* in pelvic fins. We first mined *Hand2* from two chondrichthyans, elephant shark (*Callorhinchus milii*)^22^ and little skate^23^, and used transcription factor-binding site predictions to identify putative androgen response elements (AREs; see Methods). *In silico* analyses revealed two ARE motifs (*Hand2*-ARE1 and *Hand2*-ARE2) within highly conserved regions of the 3′ untranslated region (UTR) of *Hand2* in elephant shark and in little skate (Fig. 4d,e). Comparative genomic and transcription factor binding site analyses showed conservation of these two *Hand2* AREs in all vertebrates sampled (Fig. 4e). To determine whether *Hand2*-ARE1 and *Hand2*-ARE2 can directly bind AR, we used an electrophoretic mobility assay (EMSA) with nuclear extract from AR-positive LNCaP prostate adenocarenoma cells (Fig. 4f and Methods). Labelled probes for *Hand2*-ARE1 and *Hand2*-ARE2 plus LNCaP extract formed a shifted complex, confirming DNA:protein interaction (Fig. 4f). The complex was diminished by the addition of cold competitors, and addition of AR antibody resulted in a block shift of skate and mouse *Hand2*-ARE1 and *Hand2*-ARE2, indicating direct interaction between AR and these motifs. (Fig. 4f). Taken together, these results reveal that AR has a direct input into the Shh pathway via two evolutionarily conserved *Hand2*-AREs.

## Discussion

The results presented here demonstrate that androgen signalling maintains the genetic circuit for fin development in the clasper-forming region of male pelvic fins, and that Shh alone is both necessary and sufficient for formation of clasper skeletal elements. On the basis of these data, we propose that the origin of chondrichthyan claspers resulted from acquisition of a hormonal regulatory input into the fin development circuit, and our results suggest that this may have been facilitated by evolution of AREs in the *Hand2* locus. This is the first demonstration that *Hand2* expression can be modulated by androgen, although interactions between *Hand2* and other sex steroids (for example, progesterone) are known to occur in mammals^24^. These results raise the possibility that *Hand2* could mediate androgen signalling in other developmental and disease contexts across vertebrates.

Discoveries of claspers in the most basal gnathostomes provide temporal and phylogenetic context for our analysis of clasper development in the skate model. New data and reinterpretations of placoderm fossils have cast doubt on the structural homology of chondricthyan and placoderm claspers because of their different anatomical positions^2^,^3^. Despite these topographic differences in clasper position, the molecular mechanism of clasper development identified in our study could account for clasper development in placoderms as well as elasmobranchs. Vertebrate embryos have broad fields of lateral plate mesoderm that are competent to form appendages^25^, and cellular competence to form a polarizing region and express *Shh* extends posterior to the hindlimb buds, into the anterior tail^26^,^27^. Thus, development of claspers at more posterior positions in male placoderms could have involved AR-mediated induction of *Hand2* and *Shh* in cells positioned posterior to the pelvic fins.

Internal fertilization has evolved independently in multiple gnathostome lineages, but was not stabilized in tetrapods until the *de novo* emergence of another copulatory organ, the penis. Although elasmobranch claspers are unequivocally part of the pelvic fin and, as such, are not structurally homologous to the penis, recent work shows that tetrapod hindlimbs and external genitalia arise from adjacent pools of progenitor cells^28^,^29^. Posterior pelvic fin (this study), hindlimb bud^19^ and external genital mesenchyme express AR and develop male characters in response to androgen signalling. Localization of AR to the pelvic/genital region may reflect an ancient regionalization of the lateral plate mesoderm that facilitated sexually dimorphic development of posterior appendages in vertebrates.

## Methods

### Animal husbandry and staging

Little skate (*Leucoraja erinacea*) eggs were obtained from Marine Biology Laboratory (Woods Hole, MA) and were reared in reconstituted Fluval Marine Salt, with salinity adjusted to 32 ppt at ambient temperature. Embryos were staged and sexed according to ref. 30, which is a batoid-specific modification of ref. 31. All experiments were performed in accordance with the institutional guidelines and regulations.

### Alcian blue staining

Animals younger than stage 32 were stained intact and those at stage 32 and older were skinned and eviscerated before skeletal staining with alcian blue according to published methods^32^,^33^,^34^. Briefly, embryos were fixed overnight in 4% paraformaldehyde, and then stained with 0.2% alcian blue dissolved in ethanol containing 30% glacial acetic acid (acid alcohol) for 1–3 days. Specimens were then destained in acid alcohol for 2–4 days, and then rehydrated and cleared through a graded glycerol series in 0.5% KOH before photography.

### Cloning and characterization of novel genes

Skate orthologues of *Hand2 Grem1*, *Bmp4*, *Msx2* and *Spry4* were cloned by RT–PCR using cDNA pools made from stage 31 male *L. erinacea* embryos. Primers were designed using whole-genome shotgun sequences available on NCBI. PCR products were cloned into pGEMT-EZ vector (Promega) and sequenced by Sanger sequencing in both directions. Orthology was determined by molecular phylogenetic analysis using the MrBayes programme for Bayesian inference (trees are shown in Supplementary Fig. 6).

### *In situ* hybridization

Whole-mount RNA *in situ* hybridization was performed according to published methods^35^ with the following modifications: Proteinase K concentration was 20 μg ml^−1^, KTBT buffer contained 1% Triton X-100 and alkaline phosphatase buffer (NTMT) contained 0.1% Tween-20. Colorimetric reaction was performed using BM Purple substrate (Roche). Embryos were then rinsed in PBS and post-fixed in 4% paraformaldehyde. Embryos were immersed briefly in methanol before photography.

### Bead implantations

Bead implantations were performed according to published methods^6^ with the following modifications: Heparin acrylic (Sigma) and Affigel-Blue (Bio-Rad) beads were size-selected at 250** **μm. Affigel-blue beads were soaked in SHH-N protein (human origin, R&D Systems) diluted to 1 mg ml^−1^ in PBS with 0.1% BSA. Heparin beads were soaked in cyclopamine (Toronto Research Chemicals) diluted to 4 mg ml^−1^ in DMSO. Control experiments were performed using the respective vehicle-only beads. Stage 30 embryos were dissected from their egg cases, anaesthetized and the bead was surgically implanted into the posterior region of the pelvic fin using tungsten needles. For all bead implants, *L. erinacea embryos* were sorted by sex and then randomly allocated into either control or experimental treatment groups. After manipulation, animals were returned to their tank for 24-h before tissue-harvesting for qRT–PCR, or they were allowed to develop for an additional ∼10 weeks for phenotypic analysis.

### Phenotypic analysis

A subset of animals implanted with SHH, cyclopamine and control beads were assessed for differences in proximodistal outgrowth of the post-basipterygial skeleton. Measurements were taken using the Straighten function of the NIH ImageJ software. Measurements were taken from the posterior point of the basipterygium to the most distal skeletal element present. The length of the bead-treated fin was normalized against the contralateral untreated fin in order to correct for variation in animal size. Differences in proximodistal lengths were quantified using a two-tailed Welch's *t*-test with the *α*-level set to 0.05.

### Hormone and drug treatments

11-KT and the AR antagonist flutamide (both from Sigma) were diluted to appropriate concentrations in absolute ethanol. *L. erinacea* embryos were removed from egg cases, sorted by sex, and then randomly allocated into either control or experimental treatment groups. Treated female embryos received 11-KT (0.1 μg l^−1^) and males received flutamide (1 mg l^−1^). Doses were determined in our own preliminary experiments based on published data^36^. Each biological replicate was cultured in a single 100-ml glass beaker of seawater plus either the treatment or vehicle control. Water changes and renewal of treatment/vehicle were carried out every 24 h throughout the 96-h duration of the experiment.

### qRT–PCR

Primer design was carried out using Primer Quest (IDT DNA Technologies, Supplementary Table 2). To ensure specificity of primers, a 1.5% agarose gel was run after PCR to ensure presence of a single amplicon at the desired size. In addition, a melt curve analysis yielded a single distinct peak for each set, further supporting primer specificity. For tissue collection, *L. erinacea* embryos were anaesthetized using MS-222 and their pelvic fins were quickly dissected off the body wall and immersed in RNAlater (Qiagen). Tissue samples were stored at −20 °C until RNA isolation. Total RNA was isolated from embryonic pelvic fins using RNeasy plus micro kit (Qiagen). Resultant RNA quantity was determined by Nanodrop-1000 (Thermo Scientific) and quality was assessed by using a Bioanalyzer 2100 (Agilent Technologies). Only samples yielding a 260/280 of >1.90 and RNA integrity number (RIN) of >8.0 were used in subsequent expression analyses. For analysis of expression changes in response to cyclopamine or SHH treatment, 200 ng of high-quality RNA was used for reverse transcription; the remaining experiments used 500 ng. RNA was reverse-transcribed to make cDNA using the Maxima First Strand cDNA synthesis kit (Thermo Scientific) and stored at −20 °C until use. qRT–PCR was carried out in 96-well format on a CFX96 Real Time system (Bio-Rad) using Sybr Green Master Mix (Bio-Rad) according to the manufacturer's protocol. GAPDH was used as a reference for SHH- and cyclopamine-treated embryos, and RPL8 was used as a reference for 11-KT- and flutamide-treated embryos. Reference genes were chosen based on their performance in teleosts under similar conditions^37^,^38^. Each biological replicate was assayed in triplicate to generate technical replicates. Results of qRT–PCR assays were determined using the ΔΔ*C*_t_ method^39^.

### *Hand2* mining and comparative genomics

The *Hand2* locus was identified first in *Callorhinchus milii* using NCBI, and subsequently in *L. erinacea* by mining whole-genome shotgun sequences (wgs) using *C. milli* as a query. Orthologous loci were then identified in zebrafish, coelacanth, turtle, finch, horse, mouse and human using Ensembl Genome browser. Identification of putative androgen response elements was carried out using LASAGNA^40^ and JASPAR^41^,^42^, and comparative genomic analyses were performed in VISTA^43^,^44^ with conservation parameters of min y: 60 and min ID: 65.

### Electrophoretic mobility shift assay

Single-stranded 5′ biotinylated oligonucleotides (both sense and antisense) were purchased from IDT DNA Technologies (see Supplementary Table 3). Oligos were first reconstituted in annealing buffer (10 mM Tris, pH 7.5, 50 mM NaCl and 1 mM EDTA) and then sense and antisense were annealed together by heating to 95 °C for 5 min before slowly cooling to room temperature. We utilized LNCaP nuclear extracts (Active Motif) as a source of activated androgen receptor. The gel shift was then carried out using the LightShift Chemiluminescent EMSA Kit (Pierce) with the following modifications: reaction buffer consisted of 1 × binding buffer, 5% glycerol, 5 mM MgCl_2_, 50 ng μl^−1^ poly (dI·dC) and 0.05% NP-40. All incubations were carried out on ice for 40 min, and the final reactions contained 40 fmol of biotin-labelled oligos. For competition reactions, 200-fold molar excess of unlabelled oligo was added to the reaction mixture while block-shift experiments were achieved by the addition of 2 μg of AR-N20X antibody (Santa Cruz sc-816 X). All native gels were run in ice-cold buffer for 1 h and transferred onto a Nylon+ membrane using Trans-Blot turbo system (Bio-Rad).

## Author contributions

M.J.C. conceived the project. K.L.O. performed the experiments including bioinformatic analyses. R.D.D. contributed reagents and experimental methods. All authors contributed to the data interpretation and to the writing of the manuscript.

## Additional information

**Accession codes**: Sequences of *L. erinacea* clones have been deposited in GenBank nucleotide database under accession codes KJ994797 to KJ994799, KP720625 to KP720627.

**How to cite this article:** O'Shaughnessy, K. L. *et al*. Molecular development of chondrichthyan claspers and the evolution of copulatory organs. *Nat. Commun*. 6:6698 doi: 10.1038/ncomms7698 (2015).

## Supplementary Material

Supplementary Information Supplementary Figures 1-6 and Supplementary Tables 1-3
