# Supplementary material for: Molecular development of chondrichthyan claspers and the evolution of copulatory organs
Source: Nat Commun. 2015 Apr 14;6:6698. doi: 10.1038/ncomms7698 (PMC4403318; doi:10.1038/ncomms7698)
Supplement: Supplementary Information — Supplementary Figures 1-6 and Supplementary Tables 1-3 [file ncomms7698-s1.pdf]

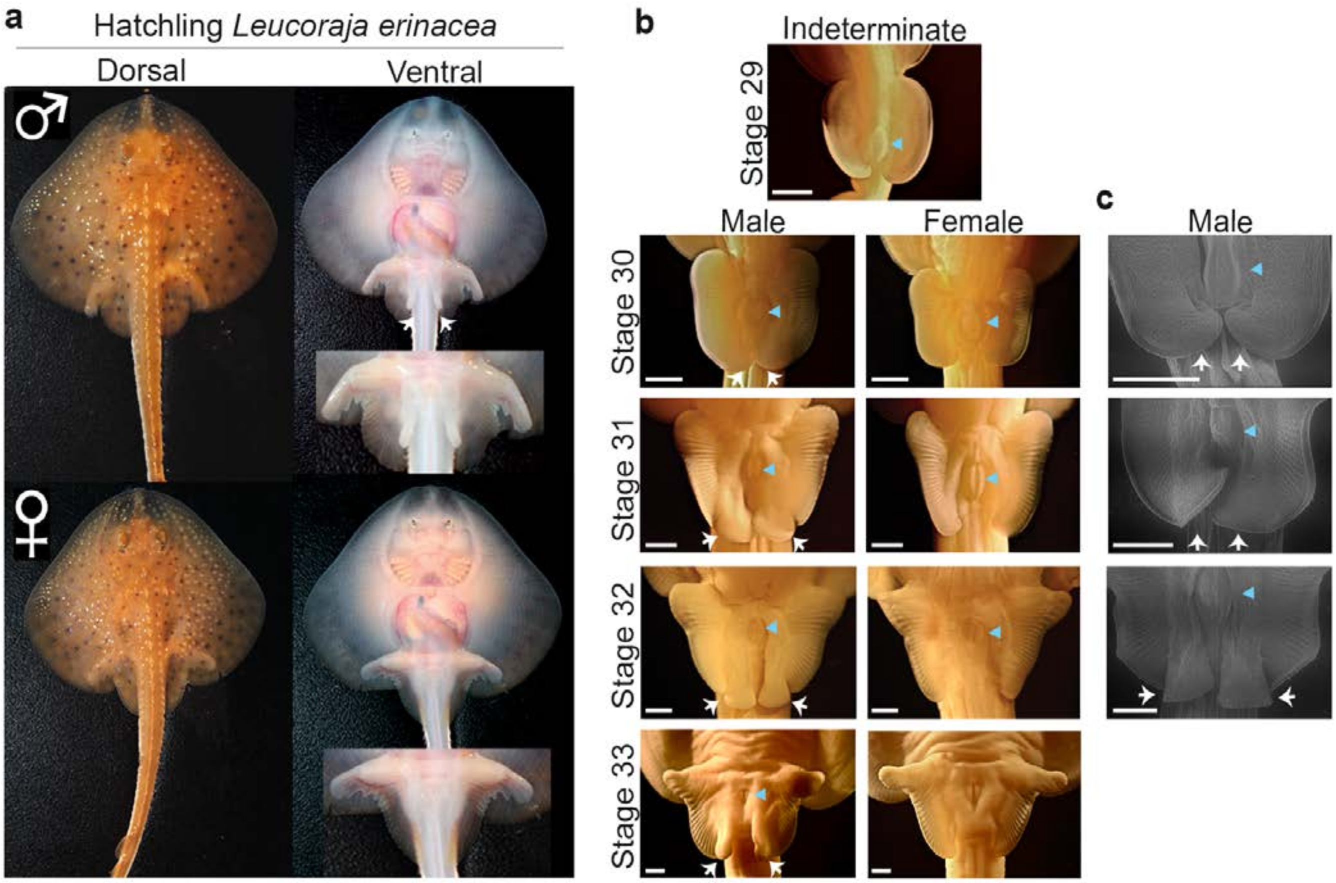

Supplementary Figure 1: Embryonic development of claspers in *L. erinacea (*little skate). a, Gross morphology of male and female hatchlings. **b,** Bouin's-fixed embryos depicting the sexually dimorphic pelvic fin development in male and female skates. White arrows mark the claspers; blue arrows mark the cloacal opening. **c**, Scanning electron micrographs showing clasper development (white arrows) in male pelvic fins at stages 30-32. In all panels the error bar represents 500 μm.

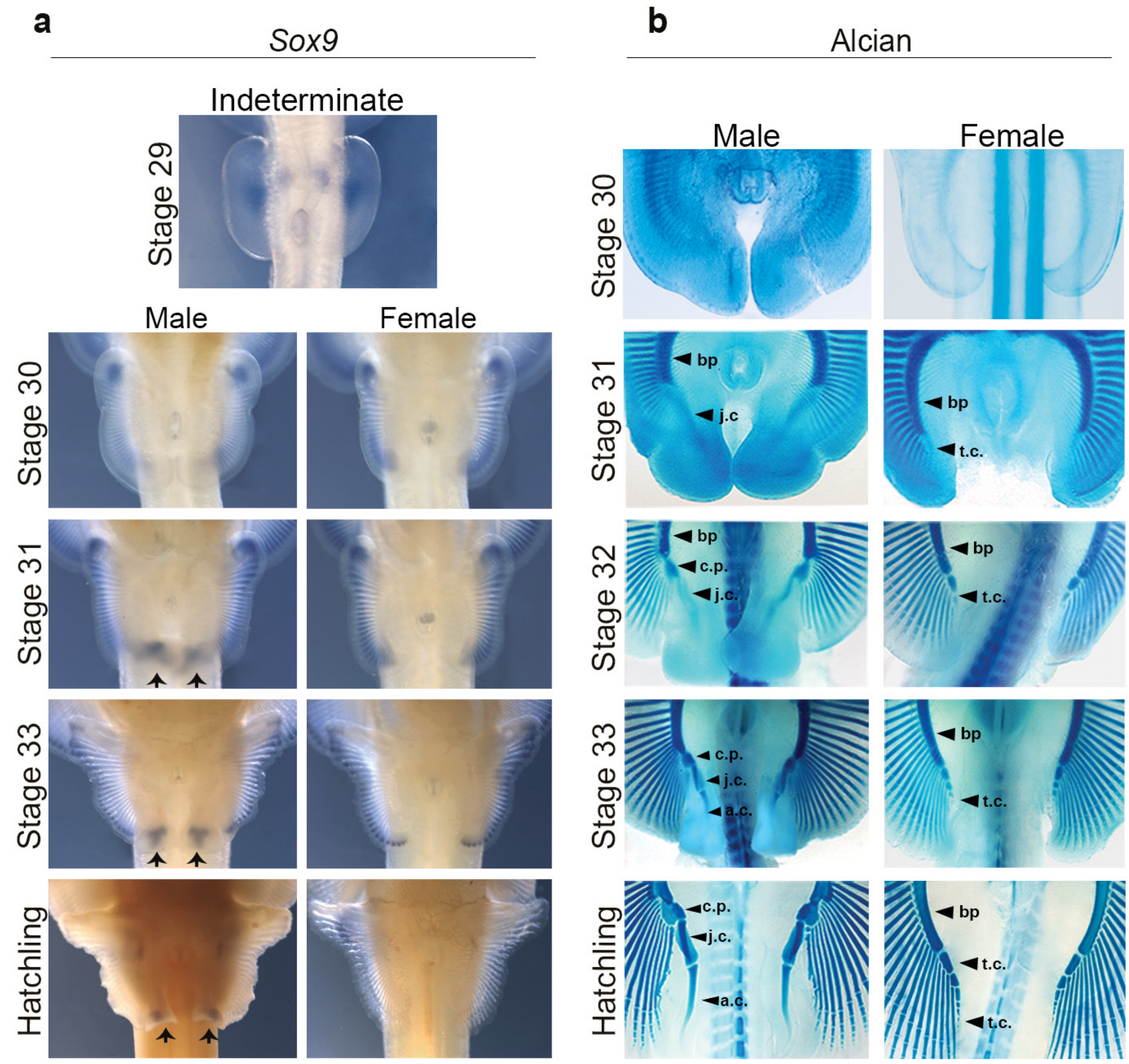

Supplementary Figure 2: Sexually dimorphic development of the pelvic fin skeleton. a, *In situ* hybridization of *Sox9* in male and female pelvic fins throughout clasper development. Beginning at stage 31, an extended posterior expression domain is apparent in male fins; posterior expression is still detected in hatchling animals. **b,** Alcian blue staining reveals differences in the development of the post-basipterygial cartilages between male and female fins. The posterior skeletal elements of males and females are labeled as follows: bp=basipterygium, t.c.=terminal cartilages, c.p.=covering plate, j.c.=junctional cartilages, a.c.=axial cartilage.

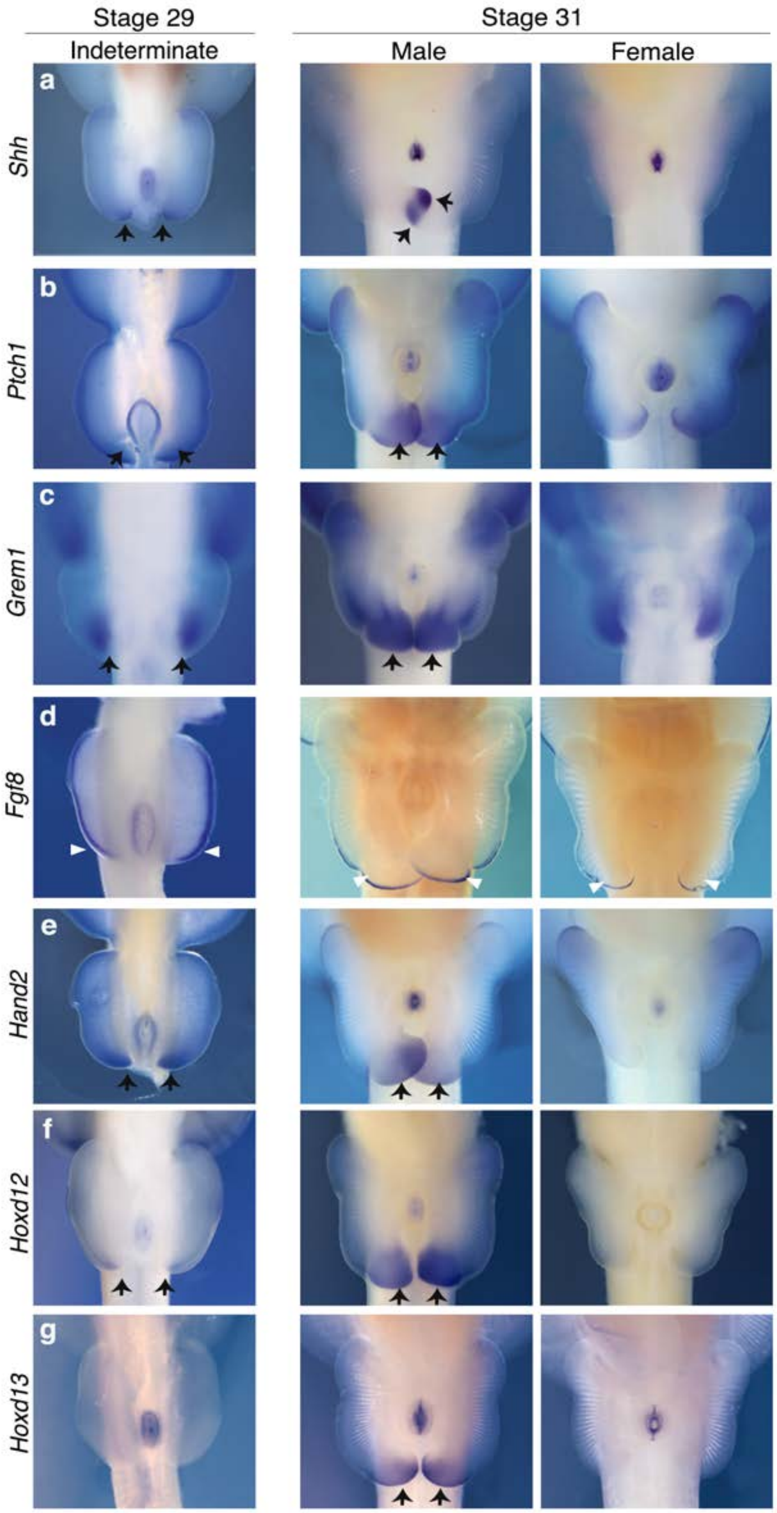

Supplementary Figure 3: Sexually dimorphic expression of genes in the fin development circuit. a-g, *In situ* hybridization of developing pelvic fins at stage 29, before sexual differentiation, and in clasper buds at stage 31. Black arrows mark mesenchymal expression and white arrows denote epithelial expression.

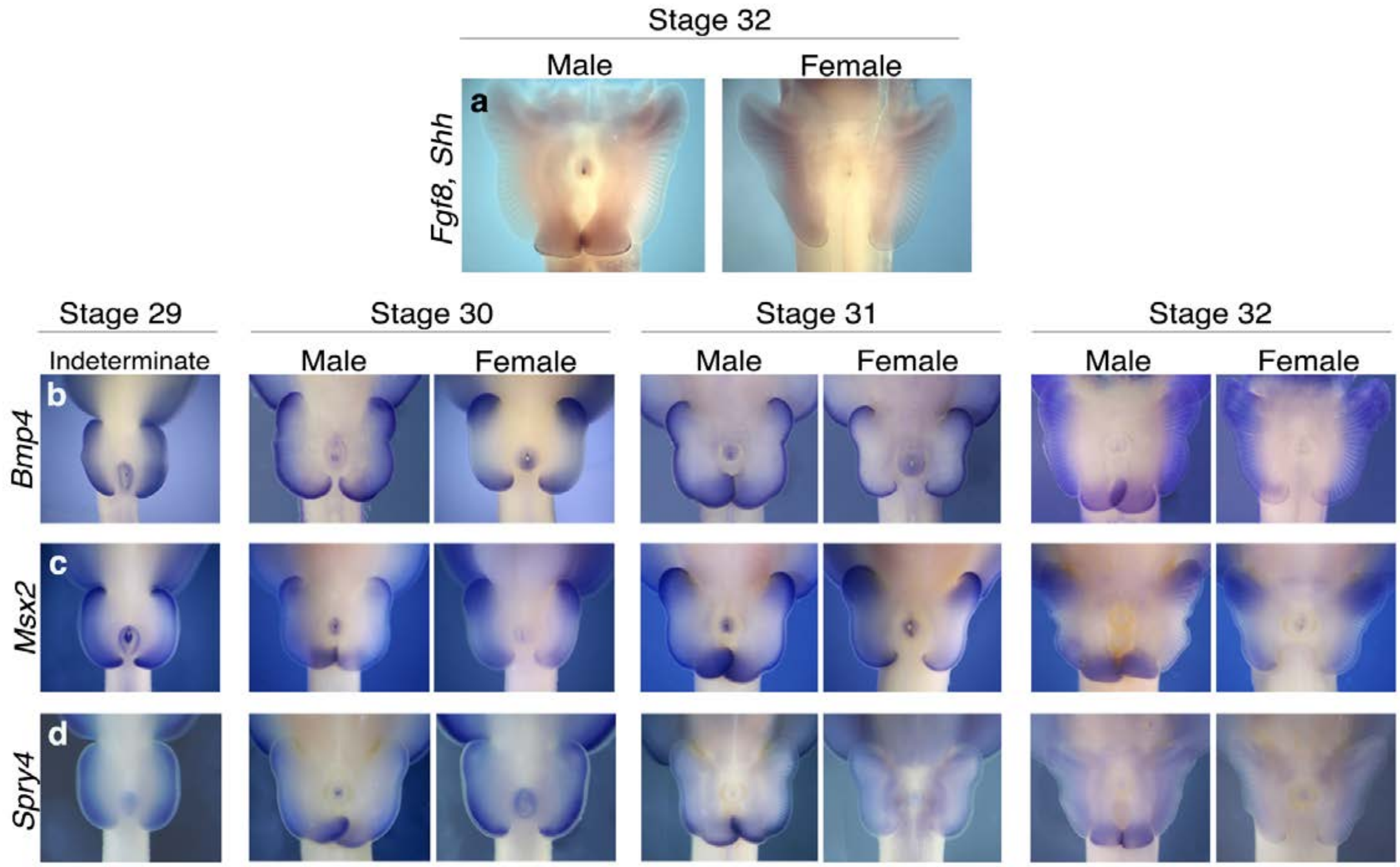

Supplementary Figure 4: The *Shh/Fgf8* feedback loop at stage 32 and *Bmp4*, *Msx2*, and *Spry4* expression patterns from stages 29-32. a, Double *in situ* hybridization in stage 32 male and female pelvic fins. *Shh* is present in the posterior margin of the male clasper bud but is undetectable in female fins; *Fgf8* is restricted to the apical ectoderm overlying the *Shh* domain in males. **b**, *In situ* hybridization of *Bmp4* show sexually dimorphic expression in the epithelium and mesenchyme of male claspers beginning at stages 31. **c,** *Msx2*, the downstream effector of Bmp4, is also sexually dimorphic beginning at stage 31. **d,** *Spry4*, a readout of Fgf signaling, shows strong posterior staining in the male clasper from stage 31. This is consistent with the timing of sexual dimorphism of *Fgf8* expression (see Supplementary Figure 3d).

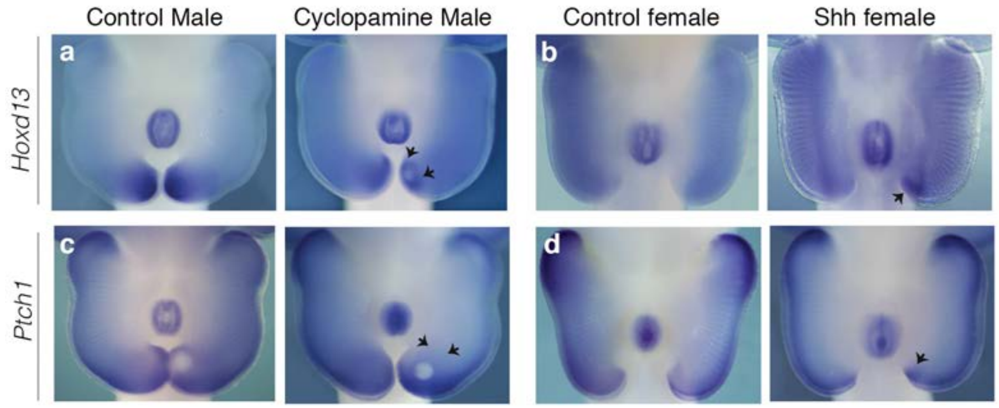

Supplementary Figure 5: Who mount *in situ* hybridizations of pelvic fins after implantation of cyclopamine or SHH beads. (a, b) The *Hoxd13* expression domain was reduced around the cyclopamine bead in male fins (a) and was upregulated around the SHH bead in female fins (b). **(c, d)** The *Ptch1* expression domain became more posteriorly restricted in male fins receiving a cyclopamine bead (c) and showed anterior expansion in female fins receiving SHH beads (d). Spatial changes in gene expression domains are subtle but consistent; quantitative changes in gene expression levels, as detected by qRT-PCR, are striking and significant (Figure 3).

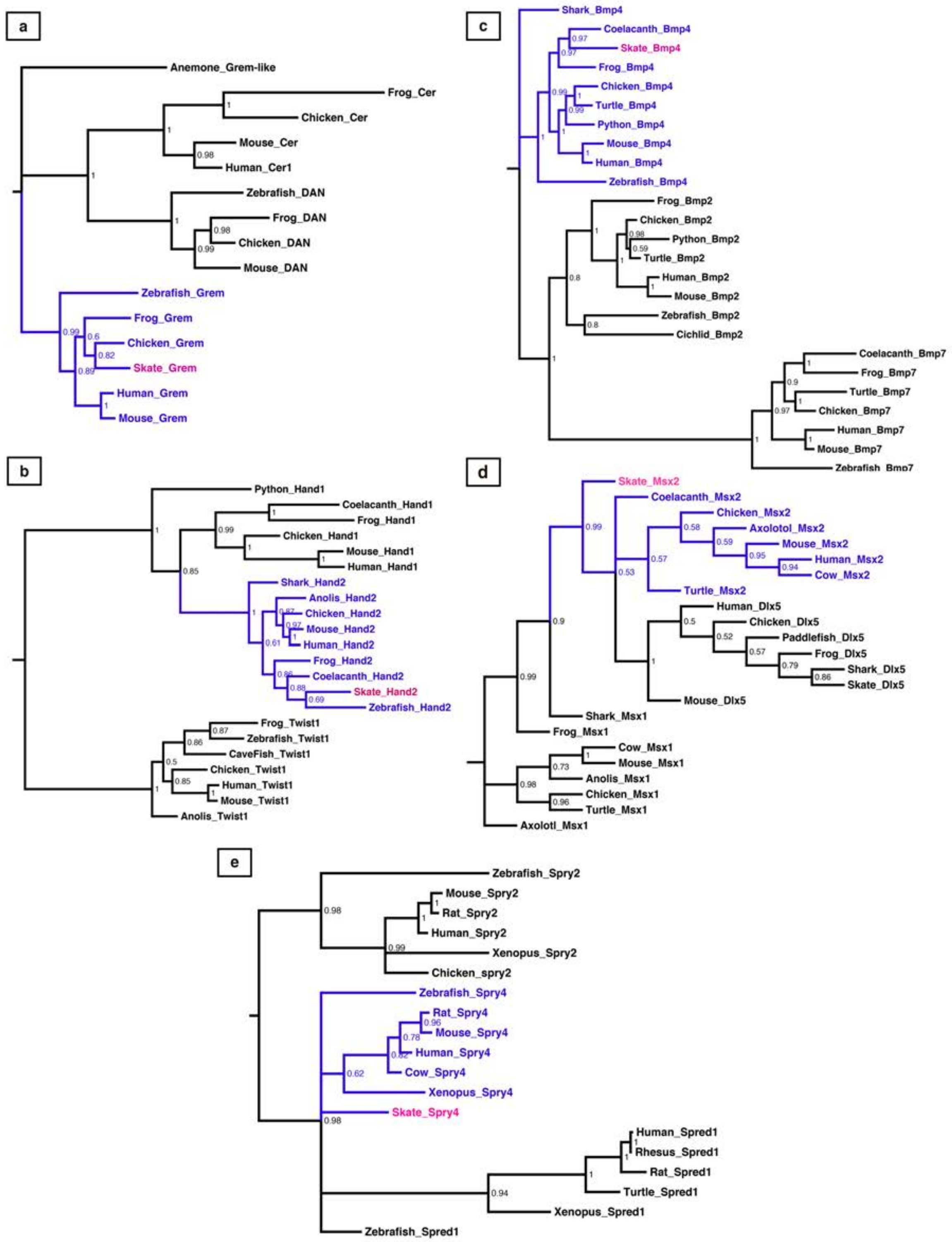

Supplementary Figure 6: Gene trees produced by Bayesian phylogenetic analyses of *Leucoraja erinacea* sequences. a-e, Nucleotide trees of *Grem1* (a), *Hand2* (b), *Bmp4* (c), *Msx2* (d), and *Spry4* (e) confirm the identity of L. *erinacea* orthologs. Bayesian phylogenetic analyses used the GTR+G nucleotide substitution model, with calculated posterior probabilities indicated at each node. The skate sequence is highlighted in pink; purple denotes orthologs in other vertebrates.

Supplementary Table 1: Table comparing developmental stages, ages, and fin morphology in *L. erinacea*. Note that days reflect development at ambient temperature.

Stage	Age (in days)	Fin morphology
29	60-70	Sexually indeterminate
30	71-80	Clasper initiation
31	81-95	Clasper bud visible
32	96-110	Continued clasper outgrowth
33	111-124	Embryonic patterning complete
Hatchling	~168	

Supplementary Table 2: qRT-PCR primers utilized in this study.

Gene	Forward	Reverse
GAPDH	5'-TGGGGCGATTCAGGAGCCCA-3'	5'-CGGGGCATCGGCACTTGGAG-3'
RPL8 (*L8*)	5'-TGTGCTGTTTGGAGGAGAAG-3'	5'-GGATTGTGGGAGATGACTGTA-3'
Shh	5'-ACAAGCAATTCATCCCGAACGTGG-3'	5'ATCAGCCTGTCAGCTCCCGTATTT-3'
Ptch1	5'-GTTTCTGGCCTGTTGTCTGCCTTGT-3'	5'-ATGCTGCCTCCCAAGCGAACATT-3'
Hand2	5'-TGCGGGATGTTGGAAGAAA-3'	5'-AAAGGAGGAACGGGAGAATAAC-3'
Hoxd12	5'-CGACAGGCTAAACCTGAGCGACC-3'	5'-GCGTTTGTTCGCGCATAACAAGTCT-3'
Hoxd13	5'-GAGTGCTGAAATGACCCAAAT-3'	5'-GAGACAGAGGGAAGGTTACAAAG-3'
Fgf8	5'-GCATGAACAAGCGAGGAAAG-3'	5'-TGCAGTGCCGTGTAGTTATT-3'
Fgfr2	5'-GGTGAACGTCGCTCTCCGCA-3'	5'-GCGCCGGTGGAGTCCGAACAT-3'
Grem1	5'-GTTCTGCTATGGCCAGTGTAA-3'	5'-GACTGTCATGGTGGTGAATCT-3'
Spry4	5'-CCGTCTCACCGTGTCATAAA-3'	5'-GGGACCATGTCTGTGTTCTT-3'
Bmp4	5'-GGGAGGTACTGCTAGTCTGATA-3'	5'-GAATTCCCGGAGCAGTTCAT-3'
Msx2	5'-CCGAGACTCAGGTGAAGATTTG-3'	5'-GGCCGACAGTTTGAGTTTCT-3'

Supplementary Table 3: Sense oligonucleotides used in EMSA. Underlined portion reflects the androgen response element (ARE) sequence, and lowercase nucleotides represent the variable 3-bp spacer.

	EMSA Sense Probe
Skate Hand2-ARE1	5bio'-AGACGTGTTG<u>AGGCCAaccAGTCCG</u>TCCCTTTGTCTT-3'
Mouse Hand2-ARE1	5bio'-AGACGTGCTG<u>TGGCCAgccTGTCCG</u>GCCTTTGGTTTT-3'
Skate Hand2-ARE2	5bio'- TATTGACAATA<u>ATTACAacgTTTACA</u>TCTCCAATAT-3'
Mouse Hand2-ARE2	5bio'-TATTGAATA<u>CTTACAatgTTTACA</u>CCTTCAATA-3'
